# Editorial: Tuberculosis: host immunity, diagnostics and therapeutics

**DOI:** 10.3389/fmmed.2025.1568256

**Published:** 2025-02-18

**Authors:** Anthony G. Tsolaki, Amy K. Barczak, Uday Kishore

**Affiliations:** ^1^ Department of Biosciences, Brunel University of London, Uxbridge, United Kingdom; ^2^ Division of Infectious Diseases, Massachusetts General Hospital, Boston, MA, United States; ^3^ Ragon Institute of MGH, MIT, Cambridge, MA, United States; ^4^ Department of Medicine, Harvard Medical School, Boston, MA, United States; ^5^ Department of Veterinary Medicine, College of Agriculture and Veterinary Medicine, United Arab Emirates University, Al-Ain, United Arab Emirates; ^6^ Zayed Centre for Health Sciences, UAE University, Al Ain, United Arab Emirates

**Keywords:** tuberculosis, drug resistance, host reponse, animal models, diagnosis, mycobacteria

Tuberculosis (TB) remains one of the world’s most significant infectious diseases, with approximately 8.7 million new cases of active disease and 1.4 million deaths annually. A third of the world’s population is estimated to harbour latent TB infection (LTBI), creating a vast reservoir for potential disease dissemination and reactivation. The complexity of host-pathogen interaction in TB, particularly during early infection and latency, continues to challenge our ability to develop effective diagnostics, vaccines, and therapeutics.

This Research Topic aimed to attract studies that would enhance our understanding of host immunity against TB and its role in both pathogenesis and protection. The submitted papers represent diverse approaches to understanding TB, from biomarker discovery and drug resistance surveillance to host genetic factors and immune cell responses in both human and bovine TB.

Rapulana et al. addressed a critical need in TB control: the accurate diagnosis of LTBI. While current diagnostic tools such as the Tuberculin Skin Test (TST) and interferon-gamma release assays (IGRAs) have served as cornerstones of LTBI diagnosis, their limitation, particularly the frequency of indeterminate results in immunosuppressed patients, highlights the need for more reliable biomarkers. Their study identified several promising candidates, with IL-2 and IP-10 showing particularly strong potential as complementary markers to existing IFN-γ assays. The high positive and negative predictive values of these markers in ESAT-6/CFP-10 stimulated plasma suggest a possible path toward more reliable LTBI diagnosis.

The growing challenge of drug resistance in TB is highlighted by Otchere et al. in their study of difficult-to-treat TB patients in Ghana. Their identification of pre-extensively drug-resistant (pre-XDR) TB cases is particularly concerning, as it signals the potential for even more challenging treatment scenarios in the future. Their finding that 58.7% of samples showed resistance to at least one drug, with 25.2% being multidrug-resistant (MDR), underscores the urgent need for enhanced surveillance and monitoring systems. This work provides valuable insights into the molecular basis of drug resistance, particularly the distribution of mutations in key resistance-associated genes.

Fang et al. contributed to our understanding of genetic susceptibility to TB by investigating polymorphisms in the Notch4 gene. Their identification of specific SNPs associated with TB susceptibility, validated across two independent cohorts, represents a significant advance in understanding host genetic factors that influence TB risk. The demonstration that Notch4 expression increases in TB patients and correlates with disease severity, coupled with their mechanistic studies showing *Mtb*-induced Notch4 expression through TLR2/P38 signalling, opens new possibilities for host-directed therapeutic approaches.

The fourth paper, by Bhat et al., provides important insights into the immune response to bovine TB, focusing on γδ T cells—a crucial bridge between innate and adaptive immunity. Their RNA-seq analysis of different γδ T cell subsets revealed preferential activation of the WC1.1+ compartment during natural *Mycobacterium bovis* infection, characterized by upregulation of genes involved in cytotoxicity, cell activation, and chemotaxis. This work enhances our understanding of the cellular immune response to mycobacterial infection and may have implications for both bovine and human TB.

**FIGURE 1:**
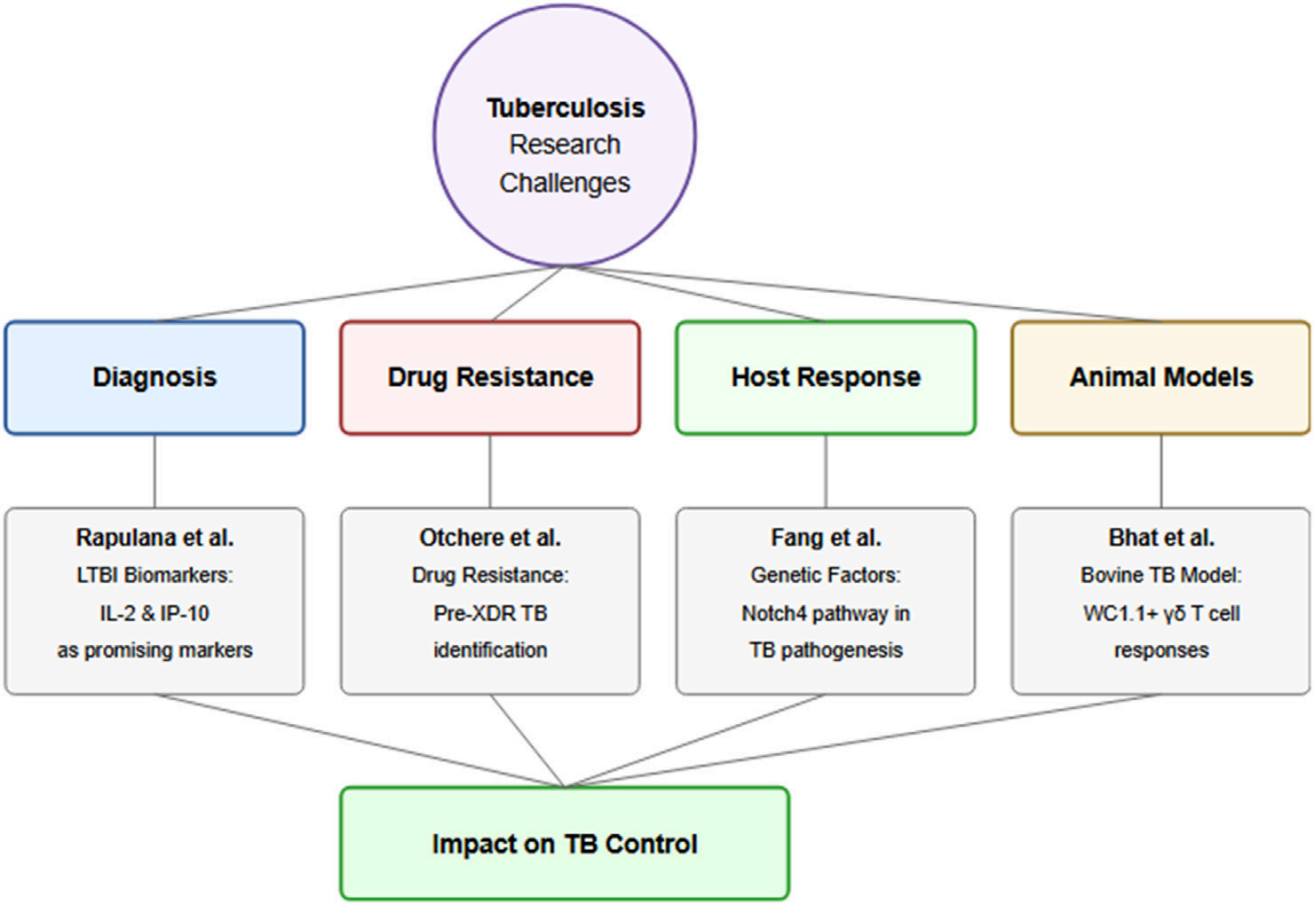
Overall contribution of papers submitted to this Research Topic.

Collectively, these papers advance our knowledge of TB in several key areas (Figure 1). First, they contribute to the development of improved diagnostic approaches, particularly for LTBI, which remains a major challenge in TB control. Second, they highlight the evolving landscape of drug resistance and the urgent need for enhanced surveillance and monitoring systems. Third, they provide new insights into host genetic factors and immune responses that influence TB susceptibility and progression, potentially opening new avenues for therapeutic intervention.

However, these studies also highlight significant gaps in our understanding. The emergence of pre-XDR TB cases emphasizes the need for new therapeutic approaches and more effective means of preventing resistance development. The complex interplay between host genetic factors and immune responses, particularly in the context of LTBI, requires further investigation. While the study of bovine TB provides valuable insights, more research is needed to understand the similarities and differences between human and bovine immune responses to mycobacterial infection.

Looking ahead, several priorities emerge from these studies. There is a clear need for:

1. Development and validation of multi-marker diagnostic approaches for more accurate diagnosis of LTBI and stratification by likelihood to progress to active TB;
2. Enhanced global surveillance systems for drug-resistant TB that both build upon and advance our molecular understanding of resistance mechanisms;
3. Development of host-directed therapeutic approaches based on our growing understanding of genetic and immunological factors that drive progression to active disease; and
4. Translation of insights from animal models to human applications

As we continue to face the challenge of TB control globally, research that bridges basic science and clinical applications, as exemplified by the papers in this Research Topic, will be crucial for developing more effective diagnostic approaches and therapeutic interventions against this persistent pathogen.

